# ADNI: Two decades of impact and the path forward

**DOI:** 10.1002/alz.083392

**Published:** 2025-01-09

**Authors:** Michael W Weiner

**Affiliations:** ^1^ University of California San Francisco (UCSF), San Francisco, CA, USA; Northern California Institute for Research & Education (NCIRE), San Francisco, CA, USA; San Francisco Veterans Administration Medical Center (SFVAMC), San Francisco, CA, CA USA

## Abstract

The Alzheimer’s Disease Neuroimaging Initiative (ADNI) has made many important contributions to the development of Alzheimer’s Disease (AD) disease modifying treatments and diagnostic biomarkers. Since its funding in 2004 by the National Institutes of Aging, the goal of ADNI has been the validation of biomarkers for AD treatment trials.

ADNI has enrolled over 2,400 participants in the USA and Canada for longitudinal clinical, cognitive, and biomarker studies. A major accomplishment is that ADNI data has been widely used for the design and of Phase 2 and 3 clinical trials, including Aducanumab, Lecanemab, and Donanemab. In addition, ADNI first demonstrated the feasibility of multisite amyloid PET scans leading to FDA approval for amyloid imaging. ADNI MRI and PET standardized protocols, widely used by academe and industry, have allowed the efficient collection of large image databases, with many thousands of MRI and PET scans. ADNI demonstrated the feasibility of multisite lumbar punctures and validated CSF amyloid and tau, leading to FDA approved CSF diagnosis. ADNI demonstrated relationships between clinical decline and amyloid, the role of tau in driving cognitive decline, showing the feasibility of prevention studies such as A4, AHEAD, and TrailblazerAlz3.

ADNI provides all de‐identified data to the scientific community without embargo through the ADNI website adni.loni.usc.edu, leading to over 5,500 publications. The ADNI public‐private partnership model for large multisite studies provided the inspiration for many studies including: DIAN, PPMI, ALL FDT, 4RTNI, LEADS and others. ADNI’s Diversity Task Force greatly increased enrollment of under‐represented participants.

Lack of inclusion of under‐represented people (especially Black and Latino adults) is a major problem for clinical trials. Our current goal is to enroll at least 50% of new participants from under‐represented groups using a culturally engaged approach with digital marketing campaigns, web‐based screening, and remote blood collection for plasma AD biomarkers. ADNI will continue to develop AD clinical trials for the future, leading to the prevention of AD symptoms and dementia.

